# Development of a Portable Taste Sensor with a Lipid/Polymer Membrane

**DOI:** 10.3390/s130101076

**Published:** 2013-01-16

**Authors:** Yusuke Tahara, Kenichi Nakashi, Ke Ji, Akihiro Ikeda, Kiyoshi Toko

**Affiliations:** 1 Graduate School of Information Science and Electrical Engineering, Kyushu University, 744 Motooka, Nishi-ku, Fukuoka 819-0395, Japan; E-Mails: k.ji@belab.ed.kyushu-u.ac.jp (K.J.); ikeda@ed.kyushu-u.ac.jp (A.I.); toko@ed.kyushu-u.ac.jp (K.T.); 2 Kyushu Institute of Technology, Fukuoka 804-8550, Japan; E-Mail: nakashi@elcs.kyutech.ac.jp

**Keywords:** taste sensor, lipid/polymer membrane, portable sensor, in-field evaluation

## Abstract

We have developed a new portable taste sensor with a lipid/polymer membrane and conducted experiments to evaluate the sensor's performance. The fabricated sensor consists of a taste sensor chip (40 mm × 26 mm × 2.2 mm) with working and reference electrodes and a portable sensor device (80 mm × 25 mm × 20 mm). The working electrode consists of a taste-sensing site comprising a poly(hydroxyethyl)methacrylate (pHEMA) hydrogel layer with KCl as the electrolyte layer and a lipid/polymer membrane as the taste sensing element. The reference electrode comprises a polyvinyl chloride (PVC) membrane layer with a small hole and a pHEMA layer with KCl. The whole device is the size of a USB memory stick, making it suitable for portable use. The sensor's response to tannic acid as the standard astringency substance showed good accuracy and reproducibility, and was comparable with the performance of a commercially available taste sensing system. Thus, it is possible for this sensor to be used for in-field evaluations and it can make a significant contribution to the food industry, as well as in various fields of research.

## 1. Introduction

In taste evaluation in the food and pharmaceutical industries, sensory tests are generally carried out by human panelists. However, this method is problematic in that it has low objectivity and reproducibility, as well as possibly imposing stress on the panelists. Use of an electronic tongue (e-tongue) or taste sensor is expected to improve such a situation. A taste sensor, *i.e.*, e-tongue with global electivity, was first proposed and patented by Toko's group in 1989, and subsequently abundant research has been performed on the application of e-tongues to the valuation of taste and quality of foods or medicines [1–4]. The taste sensing system with lipid/polymer membranes consisting of plasticizers, lipids and polyvinyl chloride can be used to evaluate the taste of foods and medicines [1–6]. The lipid/polymer membrane is the recognition element which transforms the taste information generated by the chemical substances into an electric potential change [7]. A commercially available taste sensing system consisting of taste sensors, a reference electrode, a controller and a data processing terminal has been developed by Intelligent Sensor Technology, Inc. The taste sensors are highly selective to each of five basic taste qualities and to astringency, and their characteristics are described by their global selectivity [8,9]. The five basic taste qualities are saltiness, sourness, umami, bitterness and sweetness. Additional qualities are astringency and pungency related to the sense of pain, which are considered a distinct taste quality like five basic taste qualities [10]. These qualities are also indispensable in various foods and astringency, which is sometimes considered a taste quality in the broad sense [10–12]. The instrument, however, is very heavy and expensive. If the sensing system could be reduced in size and cost to an inexpensive device capable of in-field evaluation, it would contribute significantly to the food industry and to research. Some research groups have proposed portable-type, miniaturized or disposable taste sensor systems [13–15]. However, practical implementations of these types of taste sensor, which might address the limitations of conventional laboratory analyses, have not yet been reported.

We have developed a portable taste sensor with lipid/polymer membranes which are used as sensing sites in conventional taste sensing systems [16–18]. In a previous study, we reported on a taste sensor chip with a reference electrode consisting of three layers, a poly(hydroxyethyl)methacrylate (pHEMA) hydrogel layer with KCl as the electrolyte layer, a PVC layer with 2-nitrophenyl octyl ether (NPOE) and a cellulose nitrate layer, on an Ag/AgCl electrode [16,17]. However, because of the low physical durability of this multilayered structure and the interaction between the taste substances and the chemical components of the multilayer, the electrical potential was unstable. The subjects for taste evaluation are foods and medicines which include many chemical and biological substances. Thus, the working electrode with the lipid/polymer membrane and the reference electrode need to be both physically and chemically highly durable against washing processes using washing solutions including ethanol. Moreover, the electrode potential of the reference electrode needs to be stable during the measurement period.

In this paper, we report on the successful fabrication of a new portable taste sensor using a lipid/polymer membrane. The fabricated portable taste sensor consists of a miniaturized taste sensor chip (40 mm × 26 mm × 2.2 mm) with working and reference electrodes and a portable sensor device (80 mm × 25 mm × 20 mm). The whole device is the size of a USB memory stick, making it suitable for portable use. The sensor's response to tannic acid as the standard astringency substance showed good accuracy and reproducibility, and was comparable with the performance of a commercially available taste sensing system.

## 2. Experimental Section

### 2.1. Reagents

Tetradodecylammonium bromide (TDAB) and tetrahydrofuran (THF) were purchased from Sigma-Aldrich, Inc. (St. Louis, MO, USA). Potassium chloride (KCl) and tartaric acid were purchased from Kanto Chemical Co., Inc. (Tokyo, Japan). Dioctyl phenylphoshonate (DOPP) was purchased from Dojindo Laboratories (Kumamoto, Japan). Polyvinyl chloride (PVC) was purchased from Wako Pure Chemical Industries, Ltd. (Osaka, Japan). Tannic acid was purchased from Kanto Chemical Co., Inc. (Tokyo, Japan). All aqueous solutions were prepared with distilled water.

### 2.2. Taste Sensor Chip

The fabricated sensor chip consisted of Ti/Ag electrodes patterned onto polycarbonate substrates using Ag/AgCl ink (Ag/AgCl Ink, BAS Inc., Tokyo, Japan), a strip of double-sided adhesive tape (polyimide) and a partition (polycarbonate). Polycarbonate and polyimide are well known as low-cost engineering plastics. Details on the preparation of the Ti/Ag electrodes can be found in a previous report [18].

**Figure 1.**
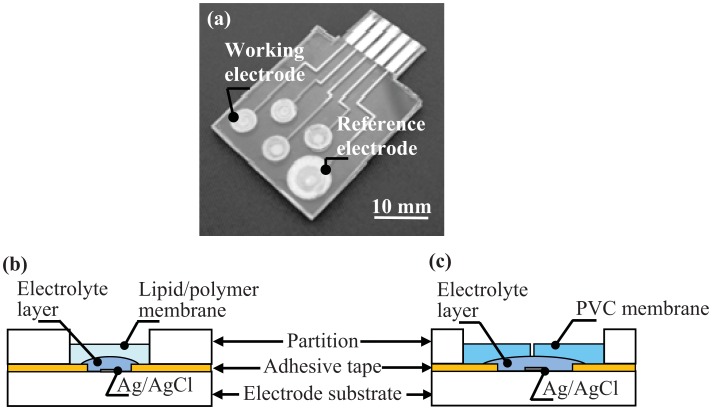
(**a**) Fabricated taste sensor chip. (**b**) Structure of the working electrode. (**c**) Structure of the reference electrode.

The working and reference electrodes were fabricated on the same side of the polycarbonate substrate (Figure 1(a)). The working electrode comprises a lipid/polymer membrane layer and a pHEMA layer with KCl as the electrolyte layer. The double-sided adhesive tape and the polycarbonate sheet have through holes with diameters of 3.0 mm and 4.5 mm, respectively, so that the electrolyte layer and lipid/polymer membrane can be set up on the electrode. The reference electrode consists of a PVC membrane layer and a pHEMA layer with KCl. The double-sided adhesive tape and the partition sheet have through holes with diameters of 6.0 mm and 7.0 mm, respectively.

The preparation of the working and reference electrodes was conducted using the following procedure. To set the electrolyte layer, a HEMA mixture solution consisting of 60 wt% hydroxyethyl methacrylate, 38 wt% ethylene glycol, 1 wt% dimethoxy-2-phenylacetophenone, and 1 wt% tetraethylene glycol dimethacrylate was dropped onto the electrode with the adhesive tape and then polymerized by UV light (2–3 mW/cm^2^) for 4 min. The volumes of these for the working and reference electrodes were 4 and 10 μL, respectively. The lipid/polymer membrane solution for the working electrode was dropped onto the electrolyte layer (Figure 1(b)). In this experiment, we used an astringency solution consisting of TDAB, DOPP, PVC and THF for the lipid/polymer membrane. It has been demonstrated that the sensor's response to astringency is in good agreement with the results of sensory tests conducted by panelists [9]. For the PVC layer for the reference electrode, PVC/THF solution was dropped onto the electrolyte layer. After drying, a small hole (φ 0.5 mm) was made through the PVC layer and the pHEMA layer of the reference electrode to form a liquid junction (Figure 1(c)).

### 2.3. Portable Taste Sensor Device and the Electronics

**Figure 2.**
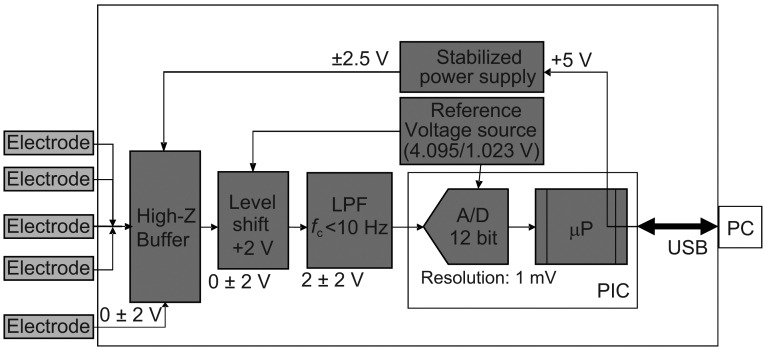
Block diagram of portable taste sensor device.

Since a commercially available taste sensing system is the size of a desktop PC and weighs more than 30 kg, this system is not suitable for in-field evaluation use. The motivation of this work is to downsize the sensor system to the size of a laptop PC for easy-to-carry and in-field applications. The developed portable taste sensor system is a combination of a USB-memory-size sensor device (80 mm × 25 mm × 20 mm) and a laptop PC for data analysis and display. Reasons for use of a PC are that the sensor device is too small to equip a sophisticated microprocessor and an LCD/FPD, and that a PC is much more powerful and flexible in terms of its performance. Figure 2 shows a block diagram of the portable taste sensor device. It consists of high impedance (high-Z) buffer amplifiers, level-shifters, low pass filters (LPFs), a PIC microcontroller with integrated 12-bit ADCs and a USB interface, and voltage regulators for internal power supply regulation. Since PIC microcontrollers are not powerful enough to do all the tasks as microprocessors can do, the PIC microcontroller does the minimal tasks such as the data acquisition control, LEDs control and communication with a laptop PC. A laptop PC connected via USB interface is responsible for all the data analysis and display. The USB interface not only communicates with the sensor device, but also provides the DC power to it, so no batteries are required. The drawback of the USB bus power is that the DC power line suffers from noises, which affect the analog circuits' performance. To reduce the power line noises, three voltage regulators regulate the analog DC power line and in-line filters/bypass capacitors suppress the noise. Due to this countermeasure, observed noise level after AD conversion is less than 1LSB and does not affect the system performance.

In the following, the electronics are explained in detail. In general, taste sensor chips have high output impedance characteristics. Therefore each channel should have a high impedance (high-Z) buffer amplifier. Taste sensor chips produce positive or negative signals relative to a reference electrode, depending on the composition of the lipid membrane. Thus, the buffer amplifiers need to handle bi-polar voltage signals (±2 V maximum). As mentioned before the sensor device is driven by USB bus power (single + 5 V supply), and buffers with a single power supply can accept only positive unipolar signals. To handle the bipolar signals, the buffers should be dual power supply. Two voltage regulators generate ±2.5 V from the USB's single + 5 V, and supply these voltages to the buffers. The input range of the buffers is ideally ±2.5 V, but due to the limitation of the input range of the buffers used, the actual input range is about ±2 V. On the other hand, the LPFs and PIC microcontroller operate on a single + 5 V power supply. In order to interface the buffers with the LPFs and the PIC microcontroller, a voltage level shift circuit is needed to convert the bipolar signal into a unipolar signal. The level shift circuit shifts the buffer output signal (0 ± 2 V) to a positive signal (2 ± 2 V), and then the level-shifted signals are fed to the LPFs to reject external noise. The cutoff frequency of the LPFs is set to 10 Hz to suppress noise from the power line (50/60 Hz). Finally, 12-bit ADCs integrated on the PIC microcontroller convert the filtered signals into the binary data (0–4,095), and send them to the PC through the USB interface. The third voltage regulator generates +4.096 V for the voltage reference of the ADCs. Therefore the system provides 1 mV/LSB resolution at best. It has four channels to accommodate up to four kinds of taste sensor chip and one reference electrode, and is suitable for in-field use.

### 2.4. The Performance of the Fabricated Reference Electrode

The reference electrode is required to have a stable electrode potential and have no response to the target substance during measurement. In order to confirm the characteristics of the reference electrode, the variation in electrode potential and its responses to target substances were evaluated. Table 1 shows the basic samples used in the experiment. Here, umami, bitterness (+), bitterness (−), astringency and sweetness samples include 30 mM KCl and 0.3 mM tartaric acid. The electrode potential of the fabricated reference electrode was evaluated in the reference solution for 2,000 s. The potential variation was measured using an electrochemical analyzer (Autolab PGSTAT302 potentiostat/galvanostat). A commercialized Ag/AgCl electrode including a saturated KCl/AgCl with a ceramic junction (RE-1C, BAS Inc.) was used as a standard electrode. The response of the fabricated reference electrode to different taste substances was measured using a SA-402B Taste Sensing System (Intelligent Sensor Technology Inc.). The fabricated reference electrode and a commercially available reference electrode were connected to this instrument. The measurement procedures for evaluating the fabricated taste sensor chip and the sensor probe were performed in the same way, following the manual. A 30% aqueous solution of ethanol containing 100 mM potassium chloride and 10 mM potassium hydroxide was used as the washing solution.

### 2.5. Performance of the Portable Taste Sensor

A fabricated taste sensor chip with a lipid/polymer membrane working electrode for measuring astringency and a reference electrode was evaluated by its response to a standard astringency substance consisting of a tannic acid solution. In this experiment, the taste sensor chip and the portable sensor were connected by leads. The signals were sent to the PC through a USB interface. The electrical potential was measured at 0.1 s intervals. The average value over a 30s period was used as the measurement value. The experimental procedure was as follows: first, the electrical potential (*V*_r_) was measured for 30 s, after the fabricated sensor chip had been immersed in the reference solution for 3 min. Secondly, the chip was immersed in the astringency sample for 2 min, after which the electrical potential (*V*_s_) was measured for 30s. Thirdly, the chip was immersed in the reference solution for 1 min, after which the electrical potential (*V*_r_′) was measured for 30s. Finally, the sensor chip was immersed in the washing solution. Here, measurements were made at room temperature and the sensor chip was immersed using a magnetic stirrer and a stirrer bar. In evaluating astringency using the commercial taste sensing system, we generally use relative values as an index of astringency and CPA (the change in the membrane potential caused by adsorption) values as an index of aftertaste [9]. The relative value (*V*_s_–V_r_) shows the difference in membrane potential between the taste sample solution (*V*_s_) and the reference solution before measurement (*V*_r_). The CPA value (*V*_r_′–*V*_r_) shows the difference in membrane potential of the reference solution after (*V*_r_′) and before (*V*_r_) measuring the sample solution. The SA 402-B Taste Sensing System was used as a conventional sensor to compare sensor performance.

## 3. Results and Discussion

### 3.1. Characteristics of the Reference Electrode

**Figure 3.**
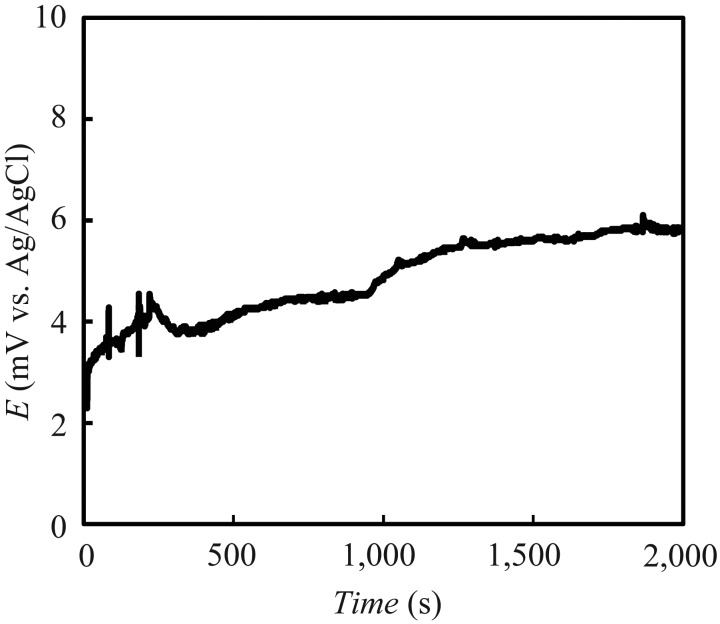
Potential variation of the fabricated reference electrode. The experiment was carried out in the reference solution.

Figure 3 shows the potential variation (*vs.* Ag/AgCl) of the fabricated reference electrode set on the sensor chip. The potential increases by approximately 4 mV over a period of 2,000 s. In our previous report [17], a reference electrode consisted of pHEMA without KCl, the potential ranged approximately 200 mV for 2,000 s. Considering the Nernst equation, this result shows that effusion of the KCl in the electrolyte layer through the hole in the PVC layer to the sample solution has occurred. However, the total measurement time for one sample from the measurement of *V*_r_ to *V*_r_′ takes approximately 270 s. Thus, it is possible for the reference electrode to be for short term disposable use. Moreover, there is the possibility of increasing the lifetime of the electrode by incubation in a saturated KCl solution, making a larger electrolyte layer or depositing a porous ceramic in the hole for the liquid junction. The performance of the reference electrode was evaluated by measuring its response to the basic taste samples. The sensor's responses (*V*_s_ – *V*_r_) to saltiness, sourness, umami, bitterness (+), bitterness (−), astringency and sweetness were −0.91 ± 0.9 (mean ± SD), 0.94 ± 0.7, 0.14 ± 0.7, 0.18 ± 0.5, −0.09 ± 0.2, −0.12 ± 0.3 mV and 0.6 ± 0.16, respectively. For the taste measurements, the reference electrode is required to not respond to the taste samples and to maintain a stationary electrical potential. The responses to the basic taste samples were all smaller than 1 mV, indicating that the fabricated reference electrode provides a stable potential without interacting with the basic taste samples and good physical durability. Thus, the characteristics of the reference electrode make it suitable for use in a portable taste sensor.

### 3.2. Performance of the Portable Taste Sensor

**Figure 4.**
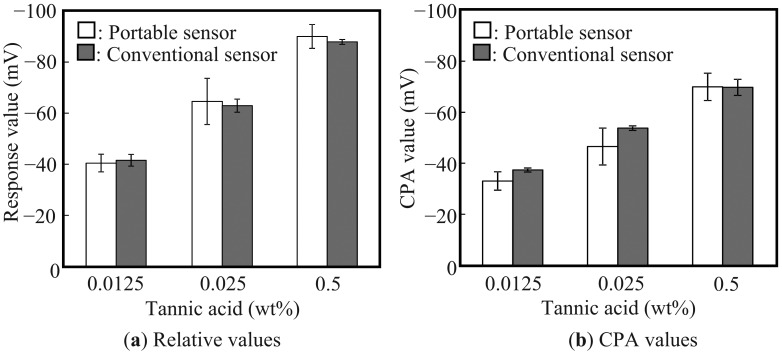
Response of the portable taste sensor to tannic acid. (**a**) Relative values. (**b**) CPA values.

The performance of the portable taste sensor for astringency was evaluated by measurements made in tannic acid. Figure 4(a) shows the relative values of the measurement. When the concentrations of the tannic acid were 0.0125, 0.025 and 0.05 wt%, the relative values of the portable taste sensor were −40.4 ± 3.5, −64.6 ± 9.0 and −90.0 ± 4.7 mV, respectively. For the conventional taste sensor the values were −41.5 ± 2.3, −62.9 ± 2.6 and −87.9 ± 0.9 mV, respectively. Figure 4(b) shows the CPA values of the measurement. The CPA values of the portable taste sensor were −33.0 ± 3.6, −46.6 ± 7.2 and −70.0 ± 5.4 mV, respectively, and those of the conventional taste sensor were −37.4 ± 0.8, −53.8 ± 0.9 and −70.0 ± 3.2 mV, respectively. These results are in good agreement, although the SDs for the portable sensor is higher than those for the conventional taste sensor. Thus, we have demonstrated that the fabricated portable taste sensor has good accuracy and reproducibility for evaluating taste in the field.

## 4. Conclusions

In this paper, we report on the development of a portable taste sensor using a lipid/polymer membrane. The sensor's responses to a standard astringency substance showed good accuracy and reproducibility, comparable with the performance of a commercial taste sensing system. Thus, the fabricated portable taste sensor has the possibility to be used for in-field evaluations and can make a significant contribution in the food industry and in various other fields of research. Additionally, it is useful to develop a more portable all-in-one sensor which does no need to be connected to a laptop PC for data analysis and display. The next step is to develop sensor chips for other taste qualities, *i.e.*, saltiness, sourness, umami, bitterness and sweetness, and carry out taste evaluation of actual foods.

## Figures and Tables

**Table 1.** Basic samples used in the taste experiments.

**Taste**	**Composition**
Reference solution	30 mM KCl, 0.3 mM tartaric acid
Saltiness	300 mM KCl, 0.3 mM tartaric acid
Sourness	30 mM KCl, 3mM tartaric acid
Umami	10 mM monosodium glutamate
Bitterness (+)	0.1 mM Quinine-HCl
Bitterness (–)	0.01 vol% iso-alpha acid
Astringency	0.05 wt% tannic acid
Sweetness	1 M Sucrose
